# “It is Easy to do Nothing and Easy to Sit Down”: Perceptions of Physical Activity and Sedentary Behaviors During Pre-retirement

**DOI:** 10.1177/07334648211062374

**Published:** 2022-02-15

**Authors:** Karl Spiteri, David R. Broom, Kate Grafton, Bob Laventure, John Xerri de Caro

**Affiliations:** 1 Faculty Research Centre for Sport, Exercise and Life Sciences, 2706 Coventry University, Coventry, UK; 2 Physiotherapy Department, St Vincent De Paul Long-Term Care Facility, Luqa, Malta; 3 School of Health & Social Care, 4547 University of Lincoln, Lincoln, UK; 4 Later Life Training; 5 Physiotherapy Department, Faculty of Health Sciences, 37563 University of Malta, Msida, Malta

**Keywords:** retirement, physical activity, successful aging, health behaviors, sedentary behavior

## Abstract

This study explored the insights of old age pre-retirement employees towards physical activity and sedentary behavior. A quota sampling of 20 participants from within the Civil Service in Malta were invited to an interview. Participants who were included met the statutory requirement for retirement within the subsequent 6 months to 1 year. Semi-structured interviews were conducted using a narrative approach. Structural narrative analysis and reflective thematic analysis were used. The story structure highlighted the significance of the individual experiences on the perceptions towards future physical activity during retirement. Two themes were identified using the thematic analysis, influencers, and perceptions. Triangulation identified that sedentary behavior was not part of the narration. The transition from work to retirement is a unique and personal experience and therefore when promoting an active lifestyle, the individual experience and past behaviors must be actively considered.

## Introduction

An aging population presents challenges because of increasing morbidity and functional impairment in a greater prevalence of the population (Teater & Chonody, 2020). Physical activity (PA) is a behavior which prevents or delays functional decline and improves quality of life (Physical Activity Guidelines Advisory Committee, 2018). Sustaining PA in older age (65 years and older), even at low intensity can decrease the risks associated with premature mortality (Hupin et al., 2015). The older age group is heterogenous in terms of the physical function of individuals as well as barriers and motivators for meeting PA recommendations for health (Morgan et al., 2019). Older adults attitudes and beliefs about PA is influenced by their culture and past experiences (Katigbak et al., 2020). Life events, such as retirement transition, have an impact on PA behavior, through an interaction between the event per se and other mediators such as past PA experience (Condello et al., 2017).

The retirement transition presents an opportunity whereby people can influence their PA behavior (Barnett et al., 2012; Spiteri et al., 2019). Retirement can act as a trigger and cause an increased awareness of the aging body. This could influence PA behavior positively or negatively (Morgan et al., 2019). For example, ageistic connotations can lead to less health promoting behavior, whilst positive attitudes towards older age can motivate to increase participation in PA (Menkin et al., 2020). The changes in PA behavior which occur when people retire indicates that there is an increase in leisure-time PA but a decrease in overall PA (Gropper et al., 2020). However, the findings are not consistent across social class and gender. Retirement is likely to result in an increase in sedentary behavior (SB) (Jones et al., 2018), especially in those who are already undertaking excessive sedentary behavior (Ter Hoeve et al., 2020). Interventions around retirement time which aimed to increase PA behavior have been inconclusive as to whether they are effective in increasing PA behavior within retiring populations (Baxter et al., 2016). One possible reason for this is that few studies have examined people’s perceptions of PA around their retirement years (Barnett et al., 2012; Gropper et al., 2020). One study explored whether retirement influences the perceptions of PA (McDonald et al., 2015). In Italy a longitudinal study examined the experience of PA across retirement in a three year-long study (Socci et al., 2021). Using a phenomenological approach, another study looked into the meaning attached to SB during the retirement transition (Eklund et al., 2021). As older adults are a heterogenous group, these studies identified the need to further research the meaning people attach to PA and SB when going through the retirement transition. Exploring pre-retirement perceptions may be useful to assist in developing effective interventions to promote a positive behavior change when people retire. Understanding the individual and the subjective experience of how the retirement process may influence their own PA and SB behaviors is required to gain an understanding of the multiple cognitive processes people are experiencing as they are preparing for retirement.

The aim of this study was to explore pre-retirement perceptions of PA and SB within the context of civil servants in Malta. Using a narrative approach to the study, of how people perceive PA and SB before retiring is well suited (Smith & Sparkes, 2009; Squire et al., 2008). The study was conducted as part of a longitudinal mixed method research using survey and interviews. People bring their own stories to life transitions (Hendricks, 2012), adopting a narrative approach allowed the researchers to consider these in relation to the retirement transition. The research questions for this study were a) What are the predictors of sedentary and PA behavior in people in the retirement transition? b) What are the differences in pre-retirement perceptions in people who are active or not active.

## Methods

A social constructionist philosophical approach was used to frame the study (Cigdem et al., 2013). A narrative methodology was identified as suitable**,** to attain the aim and remain faithful to the philosophical underpinnings of the research. As retirement is a transitional process with no fixed beginning, exploring people’s stories was deemed appropriate to learn how retirement might influence their behaviors. When using stories, people talk about their social interaction and this can be used to discover how meaning is constructed (Riessman, 2008). People interpret their experiences, and through an understanding of these it is possible to create a personal narrative (Atkinson, 1998).

### Interview Framework

Semi-structured interviews were used to collect data. Interview questions were guided by the theoretical domain framework (TDF) (Atkins et al., 2017; McGowan et al., 2020). The TDF provides an integrative summary of possible behavioral determinants based on various behavior change theories. It allowed the researcher to develop questions based on factors which could influence behavior, and not limit questioning to the researcher’s pre-conceptual ideas. All questions within the interview were open ended; the initial question allowed the participant to direct the interview according to what was important to them (supplementary file 1). During the interviews participants were guided to use their life stories. The initial question asked about the participant’s daily routine. Based on their reply further questioning took place. The interview questions were developed by KS and discussed between the researchers as they had different expertise. These were then piloted with two retiring individuals to assess their appropriateness. Due to the dual spoken languages of Maltese and English minor modifications in wording were necessary to make prompts clearer, and maintain semantic equivalence between English and Maltese (Behling & Law, 2011).

### Recruitment

The participants were selected from respondents to the associated survey. These were recruited via an open call email sent by a third party to all Civil Service employees in Malta (*n* = 881), aged 60 years and older, in September 2019. They were asked to complete a survey seeking information about their employment, the anticipated date of retirement, if they were interested *in participating* in an interview about their retirement experience and PA behavior. For the latter purpose, the Maltese version International Physical Activity Questionnaire—long version (IPAQ-long MT) (Spiteri et al., 2021)—was used. The interview participants were selected by adopting a maximum variation method based on their interest in attending an interview, as well as ensuring a distribution across civil service grades, and on the self-reported amount of PA determined by the IPAQ-long categories (low, medium, and high) (supplementary file 2). Survey respondents who indicated willingness to participate for an interview were invited to an interview at a location and time of their convenience. No time limit was set for the interviews. Purposeful sampling was used to recruit participants, this was used to get diverse views and opinions about retirement transition. The sample had a variety of people with different PA behaviors based on IPAQ categories (low, medium, and high) and job positions (non-clerical, clerical, and management). As the interviews were being conducted as a part of a 2-year longitudinal mixed methods study and due to the risk of losing participants to drop out, 20 participants were recruited for interview. After undertaking all 20 interviews, analysis was started in chronological order, and by the 16^th^ interview no new codes were emerging. This indicated that data saturation was achieved, therefore highlighting that no further participants beyond 20 were required.

### Ethics

The participants provided their written consent to respond to the online questionnaire and once again prior to the interview**,** that included permissions to record and transcribe. To maintain confidentiality and anonymity, all names reported within the study are pseudonyms whilst any description of the participants was screened to ensure that it did not allow for individual identification. Participants were also informed of their rights to withdraw from the study at any point. Ethical approval was obtained from Sheffield Hallam University Research Ethics committee reference number: ER9249191.

### Data Collection

The interviews were undertaken by one researcher (KS) to ensure consistency, over a 5-month period between September 2019 and January 2020. Interviews were conducted in either the English or Maltese language depending on the preference of the participant. Throughout the interview, notes were recorded to reflect upon later, when determining the outcomes during the data analysis and interpretation. These notes were included into a comprehensive reflective diary, which included the researcher’s thoughts before and after each interview.

### Data Analysis

The choice of analysis in narrative methods allows for flexibility (Riessman, 2008). Due to the complex nature of social phenomena, this flexibility leads to a deeper understanding (Phoenix et al., 2010). Structural narrative analysis (Bailey et al., 2013; Riessman, 2008; Smith & Sparkes, 2009) and reflexive thematic analysis using an inductive-deductive analysis (Braun & Clarke, 2006) were used jointly to analyze the data. It was decided to analyze the interview in the original language not to change meaning during the translation process (Cigdem et al., 2013). The first step within the structural narrative analysis was to produce a narrative for each participant (Riessman, 2008; Smith & Sparkes, 2009). Each of these was considered as a standalone narrative, and an individual profile was compiled (supplementary file 2). Utalising a social constructionist interpretation of their story (Cigdem et al., 2013), with every pass through the data the story structure for each participant was developed. Discussion between KS and JXDC was done on the story structure. When examining the different stories, this led to the identification of a story structural pattern when participants were discussing their PA and sedentary behavior patterns.

**Figure 1.**
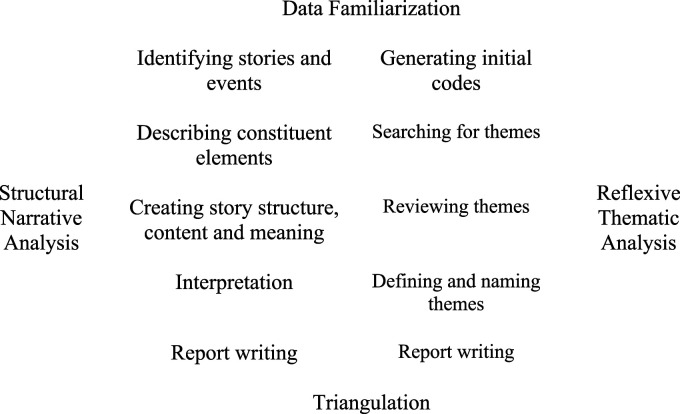
Data analysis process based on the work of Bailey et al., (2013) and Braun and Clarke (2006).

The first step of reflexive thematic analysis (Braun & Clarke, 2006), was carried out during the narrative structural analysis. Developing the individual’s story allowed for familiarization with the data. Once the narrative structural analysis was concluded, initial codes were identified and documented. The codes were then integrated into categories and another pass through the data was undertaken to ensure that the codes fitted the categories. These were then discussed between KS and JXDC, to develop overarching themes. A final pass through the data was done to check that the data fitted the identified themes. An audit trail was kept of the analysis process. Figure 1 depicts the data analysis process undertaken. To obtain data analysis triangulation, the results from both analyses were compared, and checked for congruency and differences (Leech & Onwuegbuzie, 2007). This was done by contrasting the themes identified using the thematic analysis and story structure. Data analysis was facilitated using the software program NVivo11.

### Rigor

Analysis triangulation is one of the methods used to improve trustworthiness of interpretation (Lauri, 2011). As PA is a complex behavior (Troiano et al., 2012) using multiple analyses on the same data can highlight different aspects of the phenomenon being studied (Phoenix et al., 2010). The use of an audit trail and reflective journal were used to improve confirmability of study results.

### Findings

Quotations in the participants original language are available in supplementary file 3.

The response rate to the survey was 11% (*n* = 96). Twenty participants were initially selected; one participant subsequently withdrew from the interview without giving a reason. In view of this a further respondent was selected to maintain the intended quota of 20 participants, their profile in supplementary file 2. The response rate for the interview recruitment was 95%. The age of participants ranged from 60 to 63 years. Male to female distribution was equal (*n* = 10). All worked within the Maltese civil service in diverse positions: non-clerical, clerical, and management. All participants reported to be either married or living with a partner, none of them lived alone. They all chose to be interviewed at their place of work. The interview length varied between 23 and 58 minutes.

All interviews were transcribed verbatim by the researcher and incorporated tonality details and when possible, reactions from the interview notes. This was completed to assist with the interpretation of the participants’ stories (Riessman, 2008). Prior to starting the data analysis, five interview transcripts were checked by an independent reviewer to ensure fidelity to the interviews. The transcripts were found to match the interview. 20 interviews were conducted in total, 19 in Maltese and one in English. The interviews were analyzed in the language that they were conducted to maintain semantics (Cigdem et al., 2013). Excerpts were translated into English for reporting purposes, following a discussion between KS and JXDC to ensure that the substance or general meaning was maintained. As both authors were bilingual speakers, in Maltese and English.

### Structural Narrative Analysis

**Figure 2.**
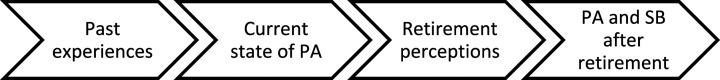
Story structure.

The structural narrative analysis approach identified patterns of how the participants regarded their future behavior patterns**,** with regards to PA and SB after their retirement from work (Riessman, 2008; Smith & Sparkes, 2009). A common structure was identified, which exemplified the way in which the participants were narrating their story. The story structure is presented in figure 2.

Two different narratives from participants were chosen. Both participants had similar roles, one viewed retirement as an opportunity to remain active and the other as a time of uncertainty**,** with long periods of extended sitting time. The story structure with specific examples is highlighted in Table 1.

**Table 1.** Story Structure with Examples.

	Albert, 61-year-old Male	Sean 61-year-old Male
Past experiences	Albert works on a shift basis; this gives him ample time to be active. Since he was young Albert was always interested in sports and felt an urge to be active. He used to cycle and play football and cycled in different periods of his life. When he was not physically active, he tried to keep himself busy by doing “active” things. Either helping out with religious activities or working in the fields. His father was regularly active as well and he got this enthusiasm for activity from him. It was passed on from father to son.	Sean is a manager, working on shifts. Throughout his life he was always aware of the importance of being active. When he was young at school, he used to play football with friends, but later family and work became a greater priority than exercise. During the summer periods, as the day is longer, he attempts to go for walks when he is off duty.
Current state of activity	Being busy is what he enjoys most. At work, the sedentary nature of his work makes him nervous and he tries to move around as much as possible.	During the week he tries to include some light walking. Being knowledgeable about the importance of being active, especially in old age, he highlights the need for him to do so. At the same time, however, his belief does not place exercise on the high priority list.
Retirement perceptions	Albert views retirement as an opportunity to continue to be active and engage in different activities; even though these might not be in the form of exercise, he is certain he will remain active.	Now that retirement is approaching, he is feeling at a loss. He is experiencing a void in his life. The lack of structured work routine is making him question his purpose in life.
Perceived PA and SB behavior after retirement	Albert has many plans and is sure that he will not spend one single day sitting down.	Sean feels that retirement is like a black hole, his prospects are not positive. Most likely he will be watching TV most of the time.

### Reflective Thematic Analysis

By the end of the thematic analysis the number of nodes identified was 337. These were integrated into six main themes**,** under the categories of Influences and Perceptions.

Themes related to Influence:

1) Learned experiences
2) Psychosocial factors shaping the retirement transition
3) The discernment aspect of retirement

Themes related to Perception:

4) Engagement in PA
5) The inevitable process of aging
6) Cognizant SB.

The development of these themes is presented in the supplementary file 4. Quotes from the interviews to support the development of the themes are present in supplementary file 5 and quotes in Maltese in supplementary file 3. Participants’ narratives are presented as supplementary file 2 to support the developed themes. Results from the reflective thematic analysis are presented in Table 2.

**Table 2.** Reflective Thematic Analysis Findings.

Theme	Quotes
1) Learned experiences	
When narrating their story, the participants frequently referred to their lived experiences, which influenced their views towards PA, health, and retirement. Life events shaped their approach towards PA, and how positive experiences, or a lack of them, impacted on their current PA behavior.	“*I lived in an era when we used to walk for our all our activities. Mummy used to take us for walks often. We used to go to school on foot…Always walking. Then it becomes part of you, you start to love walking*” [Jessie]
Participants shared their experiences of how they recalled their own parents’ experience of retirement. Overall participants used these experiences in a positive way, to have influence on the way they wanted to live their own retirement period.	“*He (in reference to his father who was also a civil servant) told me let me tell you Jason you get bored doing the same thing over and over again. That’s it, if you do your hobby every day then when you lack the desired object you would want to do more. When you have the freedom to do whatever you want you would not want to do the object anymore.*” [Jason]
2) Psychosocial factors shaping the retirement transition	
Psychosocial influences were identified as playing a pivotal role within the retirement transition. During the interview, participants spoke about different aspects which influenced the way they approached their retirement transition, and possible life adjustments thereafter. The transition from work to retirement brought on certain anxieties. Some expressed concerns about financial issues to maintain their previous lifestyle.	“*(I am) 61 and intend to think what I do ... At the moment I am doing my homework. ... I work 1 day a week and get the pension and get paid for that day, I see which one is the best option. But right now my mind is wondering*” [Chris]
Retirement was acknowledged to be an unfamiliar event. The habitual work routine that had become customary was to be replaced by something new. Participants discussed ways in which they were consciously preparing for a new life ahead. They were planning for this by making purposeful adjustments in their mindset, for the new challenges they believed that they would encounter once they retired from work.	“*Even socially if I retire from here totally I’m sure that in the morning I have to go walking with my wife at 7 in the morning she tells me lets go so if I weren’t here I would go for a walk with her at 7*” [Mike]
Retirement was viewed as a motivation and identified as an opportunity to improve on PA behaviors. This motivation was however, balanced out by the anxieties. This interplay of emotions appears to be varied. Retirement was identified as a phase during which free time was not an issue, but motivation was needed to start incorporating PA in the routine Work was regarded as an important social aspect. Even though retirement was something to look forward to, it was noted that this would result in missing out on friendships, and colleagues. The thought of losing friends made the retirement transition harder for some. This was viewed as a natural consequence of retirement, losing the social aspect of work to gain retirement.	*“(…) for me when a person stops working that is one of the problems. Because your mind starts to wonder I’m done (uhm) so it’s one of the problems …Shall I continue (working) what I shall do! (.) but that what goes through your mind at times I will say stop and sometimes I will say no. That part of it (...)”* [David]
3) Discernment aspect of retirement	
As the time for retirement approached, participants reported that they had started to take stock of their life as it had evolved until now and started to consider what they could possibly do in the next phase of their life. The life adjustments required after retirement were seen to provide new opportunities. At the same time, it was understood to be a destined change, to which they needed to adjust for. Even those who were not actively thinking about retirement, were reflecting on their life, and on what was on next.	*“...most probably 99.9% I will restart working something completely different it is a challenge for me I like to keep myself active.*” [Carmen]
It was necessary to adapt one’s life. The interruption from working within a routine, covering several hours a day needed to be filled up. It was regarded as a choice between on one hand a necessity to do something, or on the other hand do nothing. As a result of this anticipated change, it was considered necessary to plan. Whilst some developed concrete plans of what to do next, others were waiting for the post retirement period to make the necessary adaptations Most participants said that retirement presented an opportunity to increase their PA behavior, as they would have free time which could be used to be more active. Participants who considered themselves to have an active job, identified PA at work as an important contributor to their overall PA. Once work-related PA would stop, they considered it a challenge for them to replace the amount of PA at work with something else.	*“...but there comes a time when you say I want to change my routine I am old and did my part. Now I want to start another life. Sooner or later you have to start!”* [Chris]
4) Engagement in PA	
The participants’ perceptions towards PA appeared to influence their attitudes, and this in turn would impact on their level of engagement. Some viewed PA as being any form of activity, a physically active engagement, whilst other viewed PA as something specifically associated to exercise.	*“...religious commission, chairperson, teach catechesis, as in I was always active after school time (works as teacher). Now for the last 9 years, I have my son’s daughter whom I take care of and I keep myself active with her too.*” [Carmen]
Depending on their point of view, participants saw themselves as either engaging or not engaging in PA. Two other perceptions of PA were the health benefits and peer support.	*“…Frank loves to walk like me. He walks more than I do actually*.” [Josephine]
Most of the participants revealed PA as something positive which could result in varied health benefits, both physical and mental. All participants, but one, had no knowledge on the recommended levels of PA. Interestingly the participant who did know, reported to have received rehabilitation for knee pain. Sufficient PA was determined by personal perceptions, centered around being tired at the end of the day or by pushing oneself to the “personal limit.” Peer support was one of the aspects identified by most participants which was needed for engagement, or to get engaged in PA. Some participants acknowledged peer support to get going, and start a PA routine; others identified lack of support as the reason for their lack of PA.	*“...I feel stronger when I am active.”* [Mike]
5) The inevitable process of aging	
The retirement process brought about a reflection about aging in almost all participants. Two subthemes emerged: a) the change process of aging and b) self-efficacy. “Getting older” was felt to be a process of decreasing health and physical abilities.	“*No. In fact I’m going to tell you I already miss doing certain things I used to do things there (uhm) compared nowadays I slowed down and my wife tells me you are no longer the same even though you try…*” [David]
Growing older led to an expectation of increasing health problems, which would negatively impact on PA behavior due to a decrease in physical abilities. The physical ability to be active was considered to decrease with age, until a point when it had to stop.	“*And you start to see yourself getting older and you don’t want to age and are unable to do anything…*” [Carmen]
Even participants who considered themselves as active persons, reflected about the fact that PA had to stop at some point, with some hinting to a state of dependency. The recognition of aging had implications on self-efficacy. Aware of the need to adjust into a new routine, some questioned if change was still possible at their age. Changing habits was seen as a highly unlikely task. Some believed that they had enough self-determination to continue with their activity, and in doing so delay the impact of aging.	“*I mean hey, it’s something which grow with you (referring to exercise). It keeps growing in you. It is very hard to then either, because we think now I’m leaving (retiring) I can start (do exercise) eee…*.” [Sean]
6) Cognizant sedentary behavior	
SB was identified as opposite to being physically active and was associated with sitting activities. It was regarded as a “harmful” behavior which had the unintended health consequences of body stiffness or mental exhaustion when at work. However, SB was also a means to unwind after a long day at work or integrated as part of a hobby. Subthemes were identified: a) the beliefs and experiences of the effects of SB. Sitting for a long period of time was regarded as intolerable. Both during recreational activities or formal meetings. The need to stand and walk around was recognized, therefore sitting for long periods was considered restrictive. Second, b) there are unintended consequences due to the nature of activities. Sitting or lack of activity was at times an unintended consequence. Certain work practices required the person to stay seated for long periods of time, with limited possibility to move around. Participants were aware of this and voiced their concerns.	“*Work is seated for most of my day sitting down the little I get up get up let’s do it to walk a bit. I almost do it more for myself rather than for work*” [Agnes]
Some of these activities were used to relax and identified as a desired need or tiring when part of the work routine. C) a demeanor of SB and therefore striking a balance between being active and SB was important, and not in competition with each other.	*“…one I do in the morning and the other before bedtime. It does not interfere with each other. I need them both.*” [Jessie]
Engaging in SB was an easier option, being active required planning and goal setting.	“*you set goals because otherwise you end up watching television eating and instead of doing the exercise you want to do become lazier. And it is easy to do nothing and easy to sit down, easy to watch television, and very easy to eat...*” [Sean]

### Analysis Triangulation

Data analysis triangulation aimed to compare the findings obtained from two different techniques to see how much they match. If they are, it can be argued that the interpretation of the findings is more likely to be correct (Lauri, 2011). Albeit the complexity of the aging processes, using different analysis techniques can explore different aspect of the data (Phoenix et al., 2010). In this case, reflective thematic analysis and structural narrative analysis were used. The former technique attempts to identify themes from the data, while the latter attempts to identify the way the story is being told by the participants. The themes identified do fit within the story structure. Past experiences were varied and possibly influenced self-efficacy and perceptions. The current state of activity and retirement perceptions reflected the person’s life story. Projected PA and SB behavior after retirement can take place through a process of discernment, whereby psychosocial factors, aging, and experiences are taken into consideration and reflected upon to project what is next. Sedentary behavior activities did not fit within the story structure identified. Whilst PA was developed over time, SB was part of life routine. The way the two analyses merge highlights the complexity of PA behavior and the retirement transition process. The themes identified fit within different stages of the narrative structure.

## Discussion

This study is one of a few which explores the pre-retirement perception of how PA and SB might change after retirement, and in the context of Maltese civil servants is completely novel to the authors knowledge. In using two types of analysis to increase the trustworthiness of the findings, past experiences and the discernment process of retirement were identified which would likely have an influence on physical activity behavior adopted once a person retires. The pre-retirement process identified in this population fits with the transitional life course concepts (Hendricks, 2012). When analyzing the story structures, experience seemed to be one of the most influential concepts that the participants reflected upon to explain and project their life after retirement. In a recent study those who were active in the past considered PA as an aspect to maintain in retirement, whilst those who were not interested in PA were found to decrease their PA behavior (Socci et al., 2021).

It has been reported that people use their previous experiences to make sense of their current situation (Grenier, 2012). The discernment aspect is the subjective experience of people making sense of their new life realities, which they are facing. Depending on the person’s experience of their PA and SB through their life, the individual predicts their future behavior. This pattern was already identified with inauspicious pre-retirement behaviors likely to continue with the same behaviors (Ter Hoeve et al., 2020). Within this current study, participants appeared to have knowledge about the beneficial effects of PA, yet this change in behavior, from being inactive to increasing one’s activity levels, even though it might be desirable, was regarded as not easy to undertake. Participants found options such as watching television easier to engage in. Participants within the study identified SB similarly to those in the Sweden (Eklund et al., 2021). SB was identified as unhealthy compared to PA and led to poor health. There was a process of trying to be active and avoid SB. Unlike in the previous study participants did not associated SB with aging or retirement, possibly because participants were not yet retired. Discussing these concerns with health professionals might assist people to overcome them (Kava et al., 2020).

Reflecting on previous experience of PA may serve to build self-efficacy, which encourages the belief to be active. Believing in one’s ability is a motivating factor for people to engage in PA (Spiteri et al., 2019). The social cognitive theory may be used to explain some of the patterns identified within this study (Schunk & DiBenedetto, 2020). Those who had personal processes that motivated them to be active viewed retirement as an opportunity to increase their physical activity behavior. When narrating their stories, they were quick to highlight their achievements when being active. This is part of the behavior process which includes achievement, effort, and persistence. It was acknowledged that it was not always easy to remain active, even though they strived to continue. Environmental issues were important factors for setting goals such as weight control or to remain active in older age.

By employing thematic analysis and narrative structural analysis allowed to identify patterns within the participants which allows for further exploration on larger populations. However, it has to be acknowledged that the individual experiences towards retirement differ on a very subjective level (Grenier, 2012). With the same time left towards retirement the transitional process was met at different junctures. The study highlights why interventions to improve PA around this life period are important and the need to be individualized.

During the retirement transition, people consider ways to adjust to their new life, and any interventions during this time might be opportune to promote a more active lifestyle (Barnett et al., 2012; Baxter et al., 2016). Using narrative interviewing, the study was able to identify the different resources that people make use of when planning for their retirement transition; applying a resource perspective to the retirement transition explains why people might be at different stages in their adjustment and make use of the resources available to them; whether related to family, financial, socio-economical, or organizational factors (Wang et al., 2011; Wang & Shultz, 2009). All these resources might have a direct or indirect impact on retirement and the levels of activity in which people engage afterward. Considering these individualized factors and develop tailor made programs which consider participants needs can be effective in modifying PA behavior (Rowley et al., 2019). The research findings indicate towards the need to support individuals during the retirement transition**,** to help them engage in healthy behaviors. The need for peer support, through family and friends is an important factor which needs to be addressed in any intervention. Following up on person**’**s experiencing the process of retirement can allow for the identification of possible casual factors which can predict PA and SB in later life.

### Strengths and Limitations

The clear strengths of this study were the large sample size and meticulous processes by which semi-structured interviews were tested and piloted. In addition, the use of two complementary analytical approaches improved interpretation. The use of a narrative approach might have highlighted past experiences as an important aspect within retirement. However, it adds value to the person’s interpretation of how their activity levels are likely to be influenced once they retire.

A diverse participant group from different educational backgrounds and PA behavior were recruited to explore different perspectives. This was able to highlight the diverse stories of participants. Different facets of the retirement transition and PA were identified by using data analysis triangulation. However, the study results cannot be generalized to other populations and is specific to Maltese civil servants. None of the participants lived alone and the retirement transition could be experienced differently by people living alone in later life.

### Practical Application

The study highlights the importance of a lifelong perspective into the promotion of PA. Promoting of PA needs to be a positive experience so people can relate to it. When developing health promotion interventions for PA peer support systems need to be considered. The retirement transition can cause anxiety and people might need support with the adjustment. Education about the importance of being active and how to exercise at an older age is required. These applications are context specific due to the nature of the study. However, as identified within the study the retirement transition is an individualized process which highlights the importance of addressing the needs of the individual when promoting PA within this group.

### Future Research Recommendations

The study looked at the pre-retirement influences and perceptions towards PA and SB, these might change when people go through retirement and settle into their new life routine. More research is needed into how individuals experience retirement transition. Longitudinal studies are needed to assess this. The perceptions need to be evaluated in comparison with PA and SB measures and how these change over time together with their perceptions.

## Conclusion

The study adds to the body of literature on pre-retirement perceptions on PA and SB. It was able to provide an insight of Maltese Civil Servants going through the retirement transition. It highlights the subjective nature of the transition process from work to retirement. The study was able to identify the retirement transition as an adjustment period where possible intervention could take place. Within the study, activity and PA were both linked by the participants, and interventions which focus on promoting activity might also have an indirect effect on PA behavior.

## Supplemental Material

sj-pdf-1-jag-10.1177_07334648211062374 – Supplemental Material for “It is Easy to do Nothing and Easy to Sit Down”: Perceptions of Physical Activity and Sedentary Behaviors During Pre-retirement Click here for additional data file. Supplemental Material, sj-pdf-1-jag-10.1177_07334648211062374 for “It is Easy to do Nothing and Easy to Sit Down”: Perceptions of Physical Activity and Sedentary Behaviors During Pre-retirement by Karl Spiteri, David Broom, Kate Grafton, Bob Laventure, and John Xerri de Caro in Journal of Applied Gerontology

sj-pdf-2-jag-10.1177_07334648211062374 – Supplemental Material for “It is Easy to do Nothing and Easy to Sit Down”: Perceptions of Physical Activity and Sedentary Behaviors During Pre-retirement Click here for additional data file. Supplemental Material, sj-pdf-2-jag-10.1177_07334648211062374 for “It is Easy to do Nothing and Easy to Sit Down”: Perceptions of Physical Activity and Sedentary Behaviors During Pre-retirement by Karl Spiteri, David Broom, Kate Grafton, Bob Laventure and John Xerri de Caro in Journal of Applied Gerontology

sj-pdf-3-jag-10.1177_07334648211062374 – Supplemental Material for “It is Easy to do Nothing and Easy to Sit Down”: Perceptions of Physical Activity and Sedentary Behaviors During Pre-retirement Click here for additional data file. Supplemental Material, sj-pdf-3-jag-10.1177_07334648211062374 for “It is Easy to do Nothing and Easy to Sit Down”: Perceptions of Physical Activity and Sedentary Behaviors During Pre-retirement by Karl Spiteri, David Broom, Kate Grafton, Bob Laventure and John Xerri de Caro in Journal of Applied Gerontology

sj-pdf-4-jag-10.1177_07334648211062374 – Supplemental Material for “It is Easy to do Nothing and Easy to Sit Down”: Perceptions of Physical Activity and Sedentary Behaviors During Pre-retirement Click here for additional data file. Supplemental Material, sj-pdf-4-jag-10.1177_07334648211062374 for “It is Easy to do Nothing and Easy to Sit Down”: Perceptions of Physical Activity and Sedentary Behaviors During Pre-retirement by Karl Spiteri, David Broom, Kate Grafton, Bob Laventure and John Xerri de Caro in Journal of Applied Gerontology

sj-pdf-5-jag-10.1177_07334648211062374 – Supplemental Material for “It is Easy to do Nothing and Easy to Sit Down”: Perceptions of Physical Activity and Sedentary Behaviors During Pre-retirement Click here for additional data file. Supplemental Material, sj-pdf-5-jag-10.1177_07334648211062374 for “It is Easy to do Nothing and Easy to Sit Down”: Perceptions of Physical Activity and Sedentary Behaviors During Pre-retirement by Karl Spiteri, David Broom, Kate Grafton, Bob Laventure and John Xerri de Caro in Journal of Applied Gerontology

sj-pdf-6-jag-10.1177_07334648211062374 – Supplemental Material for “It is Easy to do Nothing and Easy to Sit Down”: Perceptions of Physical Activity and Sedentary Behaviors During Pre-retirement Click here for additional data file. Supplemental Material, sj-pdf-6-jag-10.1177_07334648211062374 for “It is Easy to do Nothing and Easy to Sit Down”: Perceptions of Physical Activity and Sedentary Behaviors During Pre-retirement by Karl Spiteri, David Broom, Kate Grafton, Bob Laventure and John Xerri de Caro in Journal of Applied Gerontology
