# Supplementary material for: “It is Easy to do Nothing and Easy to Sit Down”: Perceptions of Physical Activity and Sedentary Behaviors During Pre-retirement
Source: J Appl Gerontol. 2022 Feb 15;41(5):1435–44. doi: 10.1177/07334648211062374 (PMC9024021; doi:10.1177/07334648211062374)
Supplement: sj-pdf-1-jag-10.1177_07334648211062374 – Supplemental Material for “It is Easy to do Nothing and Easy to Sit Down”: Perceptions of Physical Activity and Sedentary Behaviors During Pre-retirement [file sj-pdf-1-jag-10.1177_07334648211062374.pdf]

Supplementary file 1

Interview guide

1. Can you describe your daily routine from when you wake to when you go to sleep? (Including your sedentary activities).
    1.1.1. You feel you do enough PA? Why so?
    1.1.2. When I mention a physical active person what comes to mind?
    1.1.3. What would give you the motivation to be a physically active person? Or what gives you the motivation to be a physically active person?
    1.1.4. Are there things that interfere with your physical activity?
    1.1.5. Do you predict that retirement will have an impact on your PA level? In what way?
    1.1.6. Have there been any life events which made you influence your PA
    1.1.7. Are there things that will interfere with your plan for PA after retirement?

Optional questions:

- Will retirement influence your ability to be physically active?
- How do you see your current routine change with retirement?
- Do you think retirement will help you organise your PA?
- Can you see your motivation toward PA change after retirement? If so, in what way will it change?
- What about Resources like money or access to facilities or equipment for physical activity, how this influences your physical activity?
- Your surroundings, how does it influence your PA?
- How will your significant other (family or friends) influence you during this period?
