# Supplementary material for: “It is Easy to do Nothing and Easy to Sit Down”: Perceptions of Physical Activity and Sedentary Behaviors During Pre-retirement
Source: J Appl Gerontol. 2022 Feb 15;41(5):1435–44. doi: 10.1177/07334648211062374 (PMC9024021; doi:10.1177/07334648211062374)
Supplement: sj-pdf-2-jag-10.1177_07334648211062374 – Supplemental Material for “It is Easy to do Nothing and Easy to Sit Down”: Perceptions of Physical Activity and Sedentary Behaviors During Pre-retirement [file sj-pdf-2-jag-10.1177_07334648211062374.pdf]

Supplementary file **2**

Participant's profile

Participant's pseudo name	Employment level	PA category
Agnes	Clerical	Low
Antoinette	Management 1	Low
George	Clerical	Low
Lenard	Non-clerical	Low
Sean	Management 1	Low
Vivienne	Management 1	Low
Albert	Management 1	Medium
Carmen	Management 1	Medium
Chris	Management 1	Medium
David	Non-clerical	Medium
Jason	Clerical	Medium
Jessie	Clerical	Medium
Josephine	Management 1	Medium
Josette	Clerical	Medium
Maria	Non-clerical	Medium
Raymond	Non-clerical	Medium
Claire	Management 1	High
Konrad	Management 1	High
Lilly	Clerical	High
Mike	Management 3	High

Participant's story

Lenard

Lenard is a driver and spends most of his day seated. He works on a shift basis. On his days off he is assisting his son in construction and home renovations. He worked in construction as a manual worker for 25 before becoming a driver. He was supposed to retire last year but he is working an extra year. He enjoys doing physical jobs. However, he admits this had its toll on his health, especially since he is getting older. Being active at work helps him to feel good and less stiff. In his time off he likes to walk for errands and when possible, uses public transport and walk to avoid traffic. Walking helps him to feel less stiff and him to think and relax. His PA is linked to work and there aren't any barriers to being active. He goes walking for family reasons when going out with his kids or when on short breaks.

Once he retires, given his active personality, he would like to remain active and maybe work a couple of hours a day. Retirement is seen as part of the ageing process. Due to age related changes, he cannot work as much, therefore he has to decrease his activity levels. Since he was always active, he is sure to find something to do once he retires. His ability will decrease with retirement secondary to ageing. At the same time retirement is seen as having time for self. The amount of time available to be physically active will depend on his wife's health as he has to assist her if she is unable to take care of their grandson.

David

David has been working within the civil service for the past 40 year, he will be retiring within the coming months. He works as a porter and has a lot of walking during the day to deliver letters and files through the different department. He considers himself as an active personality as PA is part of his routine. He goes walking daily with his wife and does some body weight exercises routinely as well. He is very much aware of the need to be active to remain healthy. Walking also helps him to relax from a busy day. If he gets lazy, he is scared it will be difficult for him to remain active and healthy. He acknowledged that the fact that he has no children gives him more free time and flexibility. Work gives him the push to remain active, even when it comes to the routine PA. For him retirement is a double-edged sword. It will give him more free time for self. At the same time the busy aspect of work will be lost which might hinder his motivation to be active. One of the things which David and his wife have been noticing is that as he is getting older his level of activity is decreasing. He is becoming less energetic and his initiative is decreasing. This paired with the influence of retirement is making him wonder whether retiring is the right thing to do.

Lilly

Lilly is Maltese but previously lived abroad in the UK, she has been working within the Maltese civil service for the past 7 years. She will be retiring within the coming months. She is a very active person and 'I've always' is something which she repeats to highlight that she has always been active. PA is part of who she is and part of her life. She is eager to retire to increase her activity levels. When she was in pain in the past, due to a health problem, she tried and continued to be as active as much as possible. The fact that she had to stop made her appreciate even more how important PA is for her life. For her PA is part of her routine. She sees PA as part of a healthy lifestyle and not a stand-alone thing. For her the lifestyle is passed through generations. In fact, her children are also active. She was able to adapt her PA level through her life. Exercise is alone time, even though she tries to be active with her husband. However, she prefers alone to be able to exercise at her own pace. When it comes to recreational PA, such as walks in the country she prefers to be with her husband. Knowing that one day she might be dependent on other people and unable to be active makes her 'panic'. ‘What do I do!’ her perseverance in continuing to be active is shown throughout, she managed to adapt to her circumstances to maintain a lifestyle which makes her feel healthy.

George

George works as a clerk within the civil service. He will be retiring by the end of the year. He spends most of his day working on a desk mostly being sedentary. At home he is also sedentary in his activities. He has a defeatist attitude towards exercise. He identifies himself as lazy, and unmotivated to start exercising. George is aware of the health benefits of being active even given his knee condition. However, he finds it difficult to start. He is demotivated that his activity patterns can change with retirement, since he has been inactive for a long time. So it is useless starting now. He does not feel the need to be active since he is coping in daily activities. The lack self- belief and belief that exercise will help him makes it difficult for him to see himself active. Retirement is seen as a possible opportunity due to time availability, but at the same time he has no plans or hobbies. Given his perspective he see it as unlike to change his attitude or PA level after retirement.

John

John considers himself active at work. He works as a clearer and has a lot of running about during his day. He does a lot of lifting and moving things around. When he gets home, he enjoys relaxing watching television and reading newspaper to relax. His leisure time activities are sedentary in nature. Even though he claims to like sports his activities involve occasional billiard, darts and watching football on the television. When he was young, he used to play football with friend but this only lasted a while, he prefers sedentary activity to relax. When he tries to be active, he gets lets down by feelings of breathlessness, so he does not push himself. He related this breathless to his smoking patterns which he identified as bad. Even though he claims he does not do enough PA he claims to routinely going for walks near his village and comminute regularly by foot to nearby villages. He describes himself as not fit. This could be related to his idea that PA is exercise or sports. As routine PA is not seen as part of 'PA'

He was supposed to retire this year but decided to extend for another year. He sees works as important part of his activity. He identified concerns that once he stops work, he is unlikely to compensated for this PA with his new daily routine. He did not do any plan for his retirement as yet. His aim is to use his free time in retirement to become healthier and fit. Due to work he did not have enough time to dedicate to self. He wants to stop smoking and will comminute by foot more frequently.

Albert

Albert works in management he has been working within the civil service for the past 40 years. He should be retiring in nearly a year's time. Albert is very active at work and he goes the extra mile by involving himself in extracurricular activities and social event at home. He does not like sedentary work and present work which involves movement. He has an active personality and has various hobbies which he is very passionate about. He spends most of the day being active doing things he likes when not working. Albert has been active in sport since he was young, he tried football, cycling and walking. He used to cycle to and from work until he has a near accident. Since there was an increase in traffic he stopped. The passion for cycling has been passed from generation to generation. He claims that his dad and son where both active. His type of activity varied depending on the socio and cultural context. Whatever type of activity he does he is proud of what he does. He is due to retire within the coming year however, he has certain reservations. Albert enjoys going to work and meeting people and he will miss this aspect of work. On the other hand, he is sure he will be active. since he has various hobbies, he will find things to do and will surely remain active. He does not expect to change his level of activity because he is retiring. With ageing he expect his activity to change but not to stop completely the day when he has to stop being active will happen but until then he will continue to be active and adapt.
