# Supplementary material for: “It is Easy to do Nothing and Easy to Sit Down”: Perceptions of Physical Activity and Sedentary Behaviors During Pre-retirement
Source: J Appl Gerontol. 2022 Feb 15;41(5):1435–44. doi: 10.1177/07334648211062374 (PMC9024021; doi:10.1177/07334648211062374)
Supplement: sj-pdf-3-jag-10.1177_07334648211062374 – Supplemental Material for “It is Easy to do Nothing and Easy to Sit Down”: Perceptions of Physical Activity and Sedentary Behaviors During Pre-retirement [file sj-pdf-3-jag-10.1177_07334648211062374.pdf]

Supplementary file **3** interview quotes to support theme development in original language

1) Learned experiences	"Issa trid tara il fatt ukoll li jiena trabbejt f dik l era fejn kullimkien konna immorru bil mixi. Il mummy kienet tmexxini hafna igifieri. L iskola bil mixi, konna immorru min tas sliema (.) sa paceville bil mixi. Dejjem nimxu. Imbghad tkun qisa fik, hekk fik tigi, li thobb timxi"<br>Jessie<br><br>"missieri kien ihobb mmur jonsob ezempju meta kien jahdem mal gvern mohhu ara llum hekk hu bnazzi. gieli ommi Alla jahfrila tghidlu inti mur. u ghax nofs lira. meta spicca mill irtirar l ewwel ena mar klujum, t tieni sena cempilt darba minnhom mid dar jien u irrispondih u ghedtlu ma mortx tonsob? qalli ghax a nghidlek joe qalli imbaghad tibda tixba dik ta kuljum. igifieri jekk inti taghmel hobby tieghek kuljum imbghad meta tkun nieqes l oggett mixtieq minnu iktar tkun tixtieq taghmlu. meta inti ghandek liberta li taghmlu xhin trid u hekk nahseb tibred minnu li taghmlu l oggett. u inti taghmlu x hin trid."<br>Jason<br><br>"nixtieq nkun car mieghek u emphasis. Illi meta bniedem ikun aktar attiv orrajt anki jekk ikun marid ezempju l fatt illi qed taghmel l attivita fizika inti tfisser li inti independenti. Inti independenti. Ija psychologigament ghax inti mhux the fizical only biss pscyhological inti thossok hafna hafna ahajr u fullfillled f hajtek. Jekk inti se tiddependi min kulhadd..jien ommi kienet bhali dik li nibza. Bhali attiva haga u ohra. U hadd ma kien jaghmilla bzar fuq imniehira ghax kienet intelligentissima. U din f'daqqa wahda min September sa June giet bicca laham. Motor neuron disease, attakala n nerviturni l ewwel ma wehlila ilsiena. Allura frustrazzjoni kbira imbaghad ideja u saqqa qed tifhimni. X jibqa f hajtek. Igifieri din spiccatila l attivita kollha. Allua jin ngib lila quddiemi dejjem. Li kieku ikolli nimrab bhala jien ghedtilhom ara x se taghmlu wahda injection u mmur ll hemm ghal kwiet. Ghax mhux se nissaporti. U kienet determinata li tube feeding ma tridx. X hin t tnejn gew ghax bilfors ghamlulha t tuba ghax ma tistax tiekol iktar liquid spiccat, sewwa. U x'hin qbadu t tube intilfet min sensija telqet. Ghamelt 3 tijiet u mietet. L aqwa haga. U harqa qatt ma kellha. Konna nnizluha u nohduha t toielt, mela nkella bil patella lis sptar.."<br>Mike
2) Psychosocial factors shaping the retirement transition	" *61 bi hsibni nara x naghmel igifieri jew reduced jew niehu SLSL jew nieqaf ghal kollox. BHal issa qed naghmel l homework. Pero ghadni kif cempilt lil t'hemm isfel (HR). Biex nara e jekk nahdem 2 in 3 out, igifieri jekk nahdem 30 siegha kemm naqbad paga bl allowance kollox qed nara jaqbillix hekk nkella bl SLSL dik li ghamlu issa. Tigi gurnata fil gimgha tiehu l penzjoni u tithallas ta dik l gurnata, nara x'se jaqbel. Imma bhal issa mohhi hekk qed jahden*"<br>Chris<br><br>"(…) igifieri jien taf x'nghid meta bnidem jieqaf mix xoghol dik wahda mill problema. ghax mohhok tibda tghid jien wasalt li (u:m) imma allura wahda mill problemi l bniedem jien kif nhossa jien qed nghid nieqaf mix xoghol nghid ha nkun kuntent. ha nkompli x se naghmel! (.) pero dik tkun go mohhok u gieli nghid se nieqaf u gieli nghid le. Dik wahda li (...)"<br>David

	“Riċerkatur: The fact that you won’t be coming to work, your mental ability will be influenced?<br>Lilly: It’s the mental that I’m worried about more so! because with me coming to work having less time, to look into, I go online book for information anything I don’t know Google it so to be to occupy my mind mm I’m planning this Alaskan cruise on my own rather than going to a tour.”<br>Lilly<br><br>“anki socjalment ikolli nirtira min hawn totalment nkun cert li filghodu ikolli mmur nimxi mal mara qed tifhimni fis 7 ta filghodu tghidli ejja nimxi ghax kieku ma nkunx hawn kieku nimxi maha fis 7. Allura hija importnati u. Igifieri kieku tghidli mmur fis sitta mmur. Qed nahsiba nergax nibda mmur l gym kmieni kmieni filghodu fl 4, fil 5. Ghax nohssni taghmilli iktar gid. U meta kont mmur l gym u kont mmur x xoghol mhux cajta qed nghidlek kont nidhol id dar fl 10 kemm niekol nerga nara l emails norqod u gili kont mmur fil 5 6 ta filghodu ta..”<br>Mike
3) The discernment aspect of retirement	“...most probably 99.9% li erga nibda nahdem xi haga differeti ghalija its challenge li nhobb nhobb nzomm lili attiva..”<br>Carmen<br><br>“Izjed difficli ghax issa il mara tat tifel ha jkollha tarbija iehor u il mara izzomulha. Igifieri ha jkolli noqghod mal mara mis sena id diehla.”<br>Lenard<br><br>“Riċerkatur: tahseb li l baby se jaffetwak?<br>Parteċipant: iwa, iwa<br>Riċerkatur: se jaffetwalek r routina?<br>Parteċipant: iwa iwa, ghax bi hsibni e! igifieri (hehe) l ewwel haga li ghedtilhom jien e ara ma tmorrux titilqu l baby fxi nursery. dik biss ghedtilhom jiena.<br>Riċerkatur: ok<br>Parteċipant: ghedtlihom ghax jiena naf illi baby tmur (hehe) i mean l genitui li huma l iktar irabbuhom t tfal u mhux hekk imma li titfahom f'nursery, hekk ma nistax halija jiena. forsi dik menalita tieghi ghax llum Alla hares kulhadd jaghmel kif qed nahseb jien u u kif inhua l affarijiet difficli sewwa. imma jien l affarijiet ruhi li jekk hu trid ghajnuna min ghandi se issibha. sewwa. ghal menu sakemm tieigber naqra l baby sewwa?! imbghad jaghmlu huma. imma fejn jekk issaqsuni (.) jiena bi hsibni nghin igifieri (hehe) mm”<br>Claire<br><br>“...imma jasal z zmien tghid jien irrid nibdel il il din r routina tal hajja issa kbir sehemi tajtu. issa irrid nibda l hajja l ohra. illum jew ghada trid tibdija karl! ikollok 61, u ikollok 64 l penzjoni sabiha tkun b'sahhtek. jekk tkun marid, jekk tkun marid, wara x'penzjoni hi! igifieri din nara min jibqa jahdem sa l ahhar jien ghalija ikun qed jiccewwec nifhima jien. l opinioni tieghi! igifieri jien irrid nitlaq mill penzjoni kif jien b'sahhti ha ngawdi izjed. heq mhux hekk! issa nbilli nitlaq jien b'64 u nibda kopti tugani, dari jugani, eqq titantak l awrina, ahna lirgiel. le le! e e e go sodda tkun tajjeb mhux biex tgawdi l penzjoni. jew xi naqa dementia. mhux veru.....”<br>Chris

4) Engagement in PA	"..igifieri kummisjoni djakunija, chairperson, nghalle d dutrina, igifieri dejjem kont attiva min wara l hin ta l iskola. biss issa dan l ahhar 9 snin, ghandi lit tfial tat tifla li qed nraabbija jien allura nzomm ruhi attiva biha ukoll.."<br>Carmen<br><br>"Parteċipant: emm, per ezempju qabel (.) s-sibt konna mmorru nizfnu ghax inhobb. Igifieri kieku jkolli c chance igifieri nerga' naghmilha mma r ragel heqq ma tantx ihobb, jipprova iktar affarjiet relax u hekk.<br>Riċerkatur: ok<br>Parteċipant: trid tikkuntenta lil kulhadd."<br>Maria<br><br>"Riċerkatur: din l haga hija tieghek u tar ragel?<br>Josephine: Iwa ghax Frank ihobb jimxi bhali ukoll r ragel iwa. Dak jimxi iktar minni proppjament.."<br>Josephine<br><br>"Riċerkatur: l ewwel hin minnhom semejt li tmur timxi ghalfejn taghmilha bil mixi u mhux bil karozza?<br>Parteċipant: ghax noqghod nohrog l karozza biex tmur tixtri! jekk ma ikollix xija kbira allura ghandi dak s sodisfazzjon jekk hrigt imxejt naqra forsi rajt bniedem sellimtli naqra. ifhimni jew titlaqa ma xi hadd tkun ilek ma tarah imma l bqija (.) tittawal naqa fil hwienet. biex tonfoq ghat tfal, mhux ghalija ta ghat tfal!!"<br>Vivienne<br><br>"...U inhossni aktar b'sahhti meta inkun attiv.."<br>Mike
5) The inevitable process of ageing	"le. fil fatt jien ha nghidlek naqsek diga certu affarijiet jien kont naghmel affarijiet hemm (u:m) bejn llum l gurnata naqqast u l mara tghidli. inti ma ghadekx li kont mhux billi nipprova. imma tghidli inti meta kont ta 61 mod llum 64 tghidli ma ghadejx. voldieri u vera igifieri nhossa d differenza. nhossni ghajjien u tghidli gismi. tghidli naqqas u llum l gurnata naqqast. u ghada pit ghada nissoposni nnaqas. pero ma nieqafx. (u:m) xi haga nibqa naghmel zgur.."<br>David<br><br>"Parteċipant: Yeah. I think my problem been is that if this stops I am scared that I will get depressed I don't know how to cope with it I think (.) but (.) if something happen to my brain or if something happens to me physically then I just don't know. I would just go (u:m) so there is a negative side to this as well (.) I think.<br>Riċerkatur: What do you think..<br>Parteċipant: Cos it depressed me when I was in pain with activities and it really it was to the that that. I (...) (eh) I did want to (...) I didn't want to life! I thought no! if this is (.) I don't want to (.) no! mm (..) it came to mind and that is the truth (.) yeah (.) so hey bit it will happen one day that I will be stuck may be in a wheelchair I don't know, don't know"<br>Lilly<br><br>"u tibda tara l eta gejja u ma tridx li tixjieh u li ma tiflahx taghmel xejn. jekk jista

	ikun i prolonge that process (laugh) kemm jista ikun xorta se tixjieh imma tibda attiva mhux issir. qisek cabocca fuq siggu."<br>Carmen<br><br>"Igifieri heq, hija xi haga li nahseb tikber mieghek. Tibqa tikber tibqa go fik. Difficli hafna biex imbghad la, ghax ahna nahsbu issa la nitlaq issa nibda nibda zee. Nahseb li hija xi haga li tikber go mohhok, tikber mieghek."<br>Sean<br><br>"Riċerkatur: Ghandek xi haga tghinek biex naghmel aktar attivita fizika?<br>Parteċipant: Il hsieb tahseb li inti hekk ha taghmlu gej ahjar. Jekk tahseb li inti li ha taghmel hu ahjar ghalik(.) ha taghmlu. Jekk inti mhux se taghti kas mhux se jkollok way jew tghid isma kuragg li inti ha tiprova timxi jew taghmel tip ta training. Int tghid jien li ha naghmel ghax se jghin lili. Ahna mhux jien nahseb biss bhala bnedmin meta tasal fil punt li int tghid ara x ghamilt ma iccaqlaqtx allura dak il hsieb irid jigi qabel biex inti f lahhar ma tigix fejn inti lanqas jollok sahha li taghmel dik il mixja. Trid tibda qabel. Issa dak il hsieb ghadni ghaddej. Allura nghid allura ghaddej u iz zmien ikun qieghed iqarraq bik ta.."<br>George
6) Cognizant SB	"Qatt ma mmur d dar u npoggi quddiem t television min l 4 ta wara nofs in nhar per ezempju. No way. Lanqas nissaporti (laughing) no way. Jien anki jekk nara film jekk ma nqumx 3 darbiet ma nissaportix .eee."<br>Mike<br><br>"Riċerkatur: Igifieri tuza l exerise biex jejnek mentalment u fizikament<br>Parteċipant: Ehe, ehe. U anki nkun hawn hek ta. Issa jew ghax tidra hekk dik l haga li noqod bilqeda mhux haga li togobni u nistikja. igifieri l hin kollu nivvinta x se naghmel biex nqum min hemm hemm (.) niprova.."<br>Josephine<br><br>"Nahseb jiena. Igifieri trid, trid kif taghmel, you set goals ghax inkella tispicca noqod tara it television tiekol u flok taghmel l ezercizju aktar titazzen. U facli biex biex ma taghmel xejn u facli biex toqghod bilqeda, facli biex toqghod tara t television, u facli hafna biex tiekol. Igifieri....."<br>Sean<br><br><br>"Riċerkatur: semmejt il hobbies tieghek. Li thobb taqra u hekk. Thosshom li qishom li ikun hem kompetion bejn taqrx xi ktieb jew taghmel l exercise jew tmur timxi<br>Parteċipant: Le wahda nghamila filghodu u l ohra qabel norqod. Ma tinterferix wahda ma l ohra. It tnejn bzonjuzi ghalija."<br>Jessie<br><br>"xoghol hija moghod bilqeda kwazi ghandi hafna milli gurnata bilqeda l ftit li nqum ejja nqum biex naghmel naqra dawra. kwazi kwazi iktar naghmilha ghalija milli ghax xoghol"<br>Agnes
