# Supplementary figures and images for: “It is Easy to do Nothing and Easy to Sit Down”: Perceptions of Physical Activity and Sedentary Behaviors During Pre-retirement
Source: J Appl Gerontol. 2022 Feb 15;41(5):1435–44. doi: 10.1177/07334648211062374 (PMC9024021; doi:10.1177/07334648211062374)

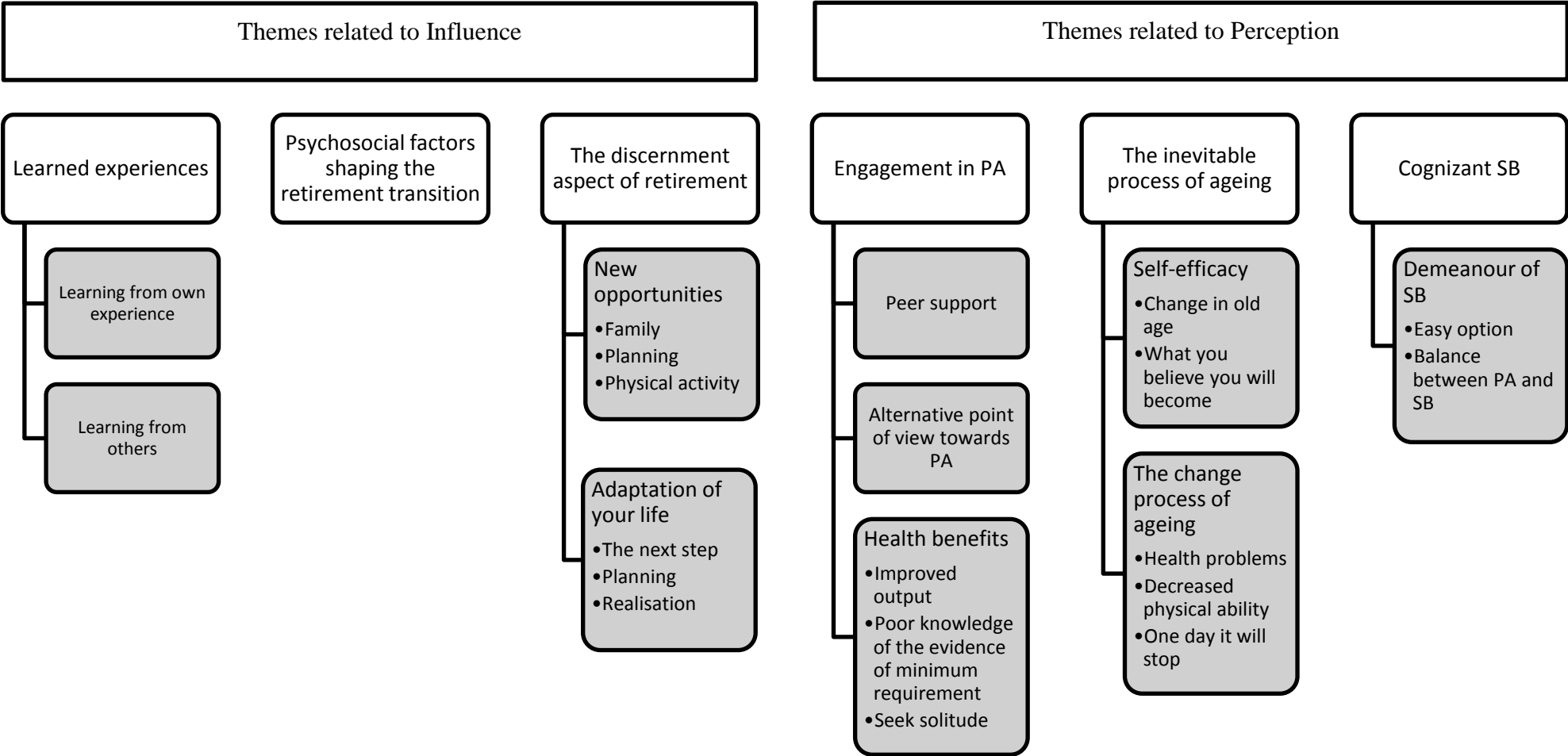

Supplement: sj-pdf-4-jag-10.1177_07334648211062374 – Supplemental Material for “It is Easy to do Nothing and Easy to Sit Down”: Perceptions of Physical Activity and Sedentary Behaviors During Pre-retirement [file sj-pdf-4-jag-10.1177_07334648211062374.pdf]
