# Supplementary material for: “It is Easy to do Nothing and Easy to Sit Down”: Perceptions of Physical Activity and Sedentary Behaviors During Pre-retirement
Source: J Appl Gerontol. 2022 Feb 15;41(5):1435–44. doi: 10.1177/07334648211062374 (PMC9024021; doi:10.1177/07334648211062374)
Supplement: sj-pdf-5-jag-10.1177_07334648211062374 – Supplemental Material for “It is Easy to do Nothing and Easy to Sit Down”: Perceptions of Physical Activity and Sedentary Behaviors During Pre-retirement [file sj-pdf-5-jag-10.1177_07334648211062374.pdf]

Supplementary file **5**: Participants' interview quotes to demonstrate theme development

1) Learned experiences	"You have to consider the fact that I lived in an era where we used to walk for our all our activities. Mummy used to make us walk a lot. We used to go to school by foot, we used to go to Sliema (.) until Paceville all on foot! Always walking. Then it becomes part of you, you start to love walking"<br>Jessie<br><br>"Dad (*refers to his father who was also a civil servant*) used to like trapping for example when he used to work with the government he was always thinking about the weather (*to go trapping*). My mum used to tell him just go.....When he retired the first year he used to go every day, once in his second year (*since his dad retired*)I called home and he took the call I told him you didn't go trapping? He told me let me tell you Jason you get bored doing the same thing over and over. That's it, if you do your hobby every day then when you lack the desired object you would want to do more. When you have the freedom to do whatever you want you would not want to do the object anymore."<br>Jason<br><br>"I want to be clear with you and emphasis. That when a person is more even if he is ill for example the fact that you are doing physical activity means you are independent. You are independent. Yes psychologically because you it is not the physical only psychologically you feel very much better and fulfilled in your life. If you are going to depend on everyone (..) My fear is that I am like my mum. She was active doing this and that like me. And no one would spit on her nose because she was very intelligent. And suddenly, in June, she because of a piece of meat. Motor neuron disease, her n nervous system got affected first lost control of her tongue. She got greatly frustrated then her hands and legs got affected. All her activity was gone. Her story is always on my mind. If I had to be in her situation I would ask for an injection and I would go quietly. Because I will not stand it. And she was determined not to get tube feeding. On Monday they came (*doctors*) because it was necessarily to do the tube because you she could not eat more liquid. And by the time they put the tube she lost consciousness. She died after 3 days. The best thing. And she never had a pressure sore. We would take her down and take her to the toilet...."<br>Mike
2) Psychosocial factors shaping the retirement transition	" (*I am*) 61 and intend to think what I do i.e. either reduced (hours) or take SLSL (redcued scheme) or quit altogether. At the moment I am doing my homework. But I just called (HR). To see if I work 2 in 3 out, that is to say if I work 30 hours how much I will get paid with allowances and all I'm check whether working SLSL is better. I work one day a week and get the pension and get paid for that day, I see which one is the best option. But right now my mind is wondering"<br>Chris<br><br>"I don't know what to say when a person stops working that is one problem. Because your mind starts to say I'm done (u:m) so it's one of the problems I feel that man says let quit my job and I'll be happy. Let me continue what I shall do! (.) but that

	what goes through your mind at times I will say stop and sometimes I will say no. That part of it (...)"<br>David<br><br>"Researcher: The fact that you won't be coming to work, your mental ability will be influenced?<br>Participant: It's the mental that I'm worried about more so! because with me coming to work having less time, to look into, I go online book for information anything I don't know Google it so to be to occupy my mind mm I'm planning this Alaskan cruise on my own rather than going to a tour."<br>Lilly<br><br>"Even socially I have to retire from here totally be sure that in the morning I have to go walking with my wife at 7 in the morning she tells me lets go so if I weren't here I would go for walk with her at 7. It's important for me. I mean if you tell me I go at six I go. I'm thinking I'll start going to the gym early in the morning at 4, at 5. Because I feel it does more good. And when I used to go to the gym and I was going to work not a joke I'm telling you I was going home at 10 as soon as I ate I would see the emails again and I would go again at 5 6 in the morning."<br>Mike
3) The discernment aspect of retirement	"...most probably 99.9% I will restart working again something completely different it is a challenge for me I like to keep myself active."<br>Carmen<br><br>"More difficult because now my sons wife is going to have another baby and my wife will be assisting her. I mean I have to stay (*at home*) with a wife as of next year."<br>Lenard<br><br>"Researcher: Do you think the baby will affect you?<br>Participant: yes, yes<br>Researcher: will r affect your routine?<br>Participant: yes yes, because I really want to! I mean (hehe) the first thing I told them doesn't leave the baby in some nursery. That's all I told them.<br>Researcher: ok<br>Participant: I told them because I know babies (hehe) I mean the parents are the most in the children upbringing and not leaving them in a nursery, I cannot accept it myself. Maybe that is my mentality because in today situation it's not possible for everyone to do as I think and and how things stand today things are very difficult. But if they want help whoever they will find it. Well. For a short period until the baby grow a bit?! But then they do whatever they want. But if they ask me (.) I intend to help (hehe) mm"

	Claire<br><br>“...but there comes a time when you say I want to change in routine I am old and did my part. Now I want to start the other life. Sooner or later you have to start! You could be 61, or 64 the beautiful of a pension is to be healthy. If I’m sick, if I’m sick, it not pension at all! In my opinion those who continue to work until the end for me are getting screwed from my understanding. It’s my opinion! I want to start retirement when I am healthy so I can enjoy it more. heq! If I leave at 64 and start with pain in my knee, my back hurts, problems with urine, we are men. No no! e e e you will be good to stay bed and you would not enjoy the pension. Or some dementia....”<br>Chris
4) Engagement in PA	“..religious commission, chair person, teach catechesis, as in I was always active after school time (*works as teacher*). Now for the last 9 years, I have my son’s daughter who i take care of and I keep myself active with her too.”<br>Carmen<br><br>“Participant: emm, for example before (.) on a Saturday we used to go dancing because I love it. I mean, if I had a chance, I'd do it again, but my husband really doesn’t like it, he prefers to relax, and so on.<br>Research: Ok<br>Participant: You have to please everyone.”<br>Maria<br><br>“Researcher: Is this something you do with your husband?<br>Participant: Yes because Frank loves to walk like me. He walks more than I do actually.”<br>Josephine<br><br>“Researcher: Before you told me you go for on foot why do it and not by car?<br>Participant: So not to get out of the car (*from the garage*) and do the shopping! if I don't have a lot of stuff to buy then I have that s satisfaction going out for a walk and maybe I meet someone to talk to talk. Or else meet with someone you haven't seen in a long time but for the rest (.) do some window shopping. Buy something for the children, not for me for children!”<br>Vivienne<br><br>“...I feel stronger when I am active.”<br>Mike
5) The inevitable process	“ No. In fact I'm going to tell you I already miss doing certain things I used to do things there (u:m) compared nowadays I

of ageing	slowed down and my wife tells me you are no longer the same even though you try. But she tells me you were 61 you were one way today you are 63 you older. So and it is true that I feel the difference. I feel tired and my body tells me. It tells me slow down and nowadays I slow down. And tomorrow it’s a pity but tomorrow I will decrease further. But I do not give up. (u: m) it is something I will keep on doing for sure.”<br>David<br><br>“Participant: Yeah. I think my problem been is that if this stops I am scared that I will get depressed i don't know how to cope with it I think (.) but (.) if something happen to my brain or if something happens to me physically then I just don’t know. I would just go (u:m) so there is a negative side to this as well (.) i think.<br>Researcher: What do you think..<br>Participant: Cos it depressed me when I was in pain with activities and it really it was to the that that. I (...) (eh) I did want to (...) I didn’t want to life! I thought no! if this is (.) I don’t want to (.) no! mm (..) it came to mind and that is the truth (.) yeah (.) so hey bit it will happen one day that I will be stuck may be in a wheelchair I don’t know, don’t know”<br>Lilly<br><br><br>“And you start to see the yourself ageing and you don't want to grow old and you can't do anything. If it can be prolong the process (laugh) as much as possible you will still age but being active you don’t become. like a coached potato.”<br>Carmen<br><br>“I mean hey, it’s something I think which grow with you (*referring to exercise*). It keeps growing in you. It is very hard to then either, because we think now I'm leaving (*retiring*) I can starts (*do exercise*) eee. I think it’s something that grows with you, in your mind.”<br>Sean<br><br>“Researcher: Do I have something which helps you to do physical activity?<br>Participant: The thought that if you do you will do better. If you think that what you do is better for you (.) You will do it. If you do not pay attention you will not have a way or say you get the courage that you will try to walk or do some kind of training. You will do if you believe it will help me. We don't just think as human beings when you get to a point where you say see what I’ve done I didn't move so that thought has to come before so that in the end you don't end up not even having the strength to do that walk. You have to start earlier. Now that thought is still ongoing (*for him*). So time goes on and I’m fooling myself.”<br>George
6) Cognizant SB	“I never go home and sit in front of the television from 4pm for example. No way. Nor do I endure (laughing) anyway. Even

if I watch a movie if I don't stand up 3 times I can't stand it."
Mike

"Researcher: What you are saying is, that you use exercise to help you mentally and physically
Participant: Yes, yes. And even here (*at work*) that is what I do. Its either because I got used to it, sitting is not something I like and able to withstand. That is it, all the time I invent what to do so I get up from sitting (.) I try."
Josephine

"I think. That is to say, you want, no matter how you do it, you set goals because otherwise you end up watching television eating and instead of doing the exercise you want to do become lazier. And it is easy to do nothing and easy to sit down, easy to watch television, and very easy to eat. I mean..."
Sean

"Researcher: You mentioned your hobbies. That you loved to reads and so on. Do you that there is a competition between reading a book or doing exercise or going for a walk.
Participant: No, one I do in the morning and the other before bed time. It does not interfere with each other. They are both need for my end."
Jessie

"Work is seated I have most of my day sitting down the little I get up get up let's get up and walk a bit. I almost do it more for myself than for work"
Agnes
