# Supplementary material for: “It is Easy to do Nothing and Easy to Sit Down”: Perceptions of Physical Activity and Sedentary Behaviors During Pre-retirement
Source: J Appl Gerontol. 2022 Feb 15;41(5):1435–44. doi: 10.1177/07334648211062374 (PMC9024021; doi:10.1177/07334648211062374)
Supplement: sj-pdf-6-jag-10.1177_07334648211062374 – Supplemental Material for “It is Easy to do Nothing and Easy to Sit Down”: Perceptions of Physical Activity and Sedentary Behaviors During Pre-retirement [file sj-pdf-6-jag-10.1177_07334648211062374.pdf]

Consolidated criteria for reporting qualitative studies (COREQ): 32-item checklist

No	Item	Guide questions/description
**Domain 1: Research team and reflexivity**		
Personal Characteristics		
1. Karl Spiteri (KS)	Interviewer/facilitator	Which author/s conducted the interview or focus group?
2. KS M.Sc.<br>Reading for a PhD	Credentials	What were the researcher's credentials? *E.g. PhD, MD*
3. Senior allied health practitioner physiotherapists. Lead service for older persons	Occupation	What was their occupation at the time of the study?
4. Male	Gender	Was the researcher male or female?
5. training in qualitative research methods	Experience and training	What experience or training did the researcher have?
Relationship with participants		
6. Two of the participants were known to the researcher. The rest were not.	Relationship established	Was a relationship established prior to study commencement?
7. Information on the interviewer was presented in	Participant knowledge of the interviewer	What did the participants know about

No	Item	Guide questions/description
the information sheet. Participants were aware that researcher was a public servant.		the researcher? e.*g. personal goals, reasons for doing the research*
8. Potential bias is that KS is a physiotherapist with an interest in older adults and a public servant.	Interviewer characteristics	What characteristics were reported about the interviewer/facilitator? e.g. *Bias, assumptions, reasons and interests in the research topic*
**Domain 2: study design**		
Theoretical framework		
9. Narrative methodology.	Methodological orientation and Theory	What methodological orientation was stated to underpin the study? *e.g. grounded theory, discourse analysis, ethnography, phenomenology, content analysis*
Participant selection		
10. Purposive sample was used based on the results of a survey.	Sampling	How were participants selected? *e.g. purposive, convenience, consecutive, snowball*
11. Email for the initial survey and then followed up by telephone call.	Method of approach	How were participants approached? e.*g. face-to-face, telephone, mail, email*

No	Item	Guide questions/description
12. Twenty	Sample size	How many participants were in the study?
13. One participant decided not to participant in the interview as initially showing interest. Reason for refusal was that the participant did not enjoy talking about his experiences.	Non-participation	How many people refused to participate or dropped out? Reasons?
Setting		
14. workplace of participants	Setting of data collection	Where was the data collected? e*.g. home, clinic, workplace*
15. Interviews were carried out in a private room or office of the participants alone.	Presence of non-participants	Was anyone else present besides the participants and researchers?
16. The age range for participants was 60 to 63. 10 participants were male and 10 females. Interviews took place between September 2019 and January 2020	Description of sample	What are the important characteristics of the sample? *e.g. demographic data, date*
Data collection		
17. The interview guide was pilot tested. The interview guide was included as supplementary material.	Interview guide	Were questions, prompts, guides provided by the authors? Was it pilot tested?
18. No	Repeat interviews	Were repeat interviews carried out? If yes, how

No	Item	Guide questions/description
		many?
19. Audio recording was done using a Dictaphone.	Audio/visual recording	Did the research use audio or visual recording to collect the data?
20. Field notes were made prior, during and after the interviews.	Field notes	Were field notes made during and/or after the interview or focus group?
21. The interview length varied between 23 and 58 minutes.	Duration	What was the duration of the interviews or focus group?
22. Yes, it was reached after the 16th interview	Data saturation	Was data saturation discussed?
23. Five interview transcripts were checked by an independent reviewer to ensure fidelity to the interviews.	Transcripts returned	Were transcripts returned to participants for comment and/or correction?
**Domain 3: analysis and findings**		
Data analysis		
24. One	Number of data coders	How many data coders coded the data?
25. Yes, this was presented in method section and supplementary files.	Description of the coding tree	Did authors provide a description of the coding tree?

No	Item	Guide questions/description
26. Themes were derived from the data	Derivation of themes	Were themes identified in advance or derived from the data?
27. Nvivo 11	Software	What software, if applicable, was used to manage the data?
28. No	Participant checking	Did participants provide feedback on the findings?
Reporting		
29. Quotation were presented in original language and translated into English when necessary. The quotes were pseudonymised.	Quotations presented	Were participant quotations presented to illustrate the themes / findings? Was each quotation identified? e*.g. participant number*
30. Yes	Data and findings consistent	Was there consistency between the data presented and the findings?
31. Yes, they were presented in findings section and in figure format.	Clarity of major themes	Were major themes clearly presented in the findings?
32. Discussion of minor themes were discussed in the findings section and in figure format.	Clarity of minor themes	Is there a description of diverse cases or discussion of minor themes?
